# A first molecular characterization of the scorpion telson microbiota of *Hadrurus arizonensis* and *Smeringurus mesaensis*

**DOI:** 10.1371/journal.pone.0277303

**Published:** 2023-01-17

**Authors:** Christopher Shimwell, Lauren Atkinson, Matthew R. Graham, Barbara Murdoch

**Affiliations:** Department of Biology, Eastern Connecticut State University, Willimantic, CT, United States of America; East Carolina University, UNITED STATES

## Abstract

Scorpions represent an ancient lineage of arachnids that have radiated across the globe and are incredibly resilient—since some thrive in harsh environments and can exist on minimal and intermittent feedings. Given the emerging importance of microbiomes to an organism’s health, it is intriguing to suggest that the long-term success of the scorpion bauplan may be linked to the microbiome. Little is known about scorpion microbiomes, and what is known, concentrates on the gut. The microbiome is not limited to the gut, rather it can be found within tissues, fluids and on external surfaces. We tested whether the scorpion telson, the venom-producing organ, of two species, *Smeringurus mesaensis* and *Hadrurus arizonensis*, contain bacteria. We isolated telson DNA from each species, amplified bacterial 16S rRNA genes, and identified the collection of bacteria present within each scorpion species. Our results show for the first time that telsons of non-buthid scorpion species do indeed contain bacteria. Interestingly, each scorpion species has a phylogenetically unique telson microbiome including Mollicutes symbionts. This study may change how we view scorpion biology and their venoms.

## Introduction

Scorpions are an ancient lineage that originated over 400 million years ago, compared to a mere 0.2–1.5 million years for humans [1, 2]. Scorpions are divided into 2 major groups, Buthida and Iurida, that are further subdivided into 22 families. Given the emerging importance of microbiomes to an organism’s health [3–5], it is intriguing to suggest that the antiquity of scorpions and their success in colonizing terrestrial environments around the globe may be linked to their microbial symbionts [6, 7]. Although investigations of bacterial symbionts in scorpions are rare, a collection of important information is beginning to emerge. Early efforts isolated DNA from a variety of tissues or whole organisms as a PCR template, using primers for single or multiple genes that were specifically targeted to a single bacterial genus. Sanger sequencing identified the PCR targets. Using this approach, *Wolbachia* has been reported in scorpions representing families Buthidae [8], Hemiscorpiidae [9], and Scorpionidae [10]. However, a screening of common arachnid endosymbionts, specifically *Wolbachia*, *Cardinium*, *Rickettsia* and *Spiroplasma*, yielded no positive results in 61 samples of the scorpion family Vaejovidae, with a cautionary note regarding off-targets of some primers [11]. Although these studies contributed to our knowledge of the distribution of a single or limited number of bacterial genera in scorpion tissues, a broader survey of taxa was needed.

To acquire a wider-ranging survey of bacterial taxa, subsequent studies used PCR primers that targeted the 16S rRNA gene of numerous bacteria, followed by subcloning into plasmid vectors and Sanger sequencing. Intermediate steps designed to reduce the number of duplicate clones sequenced included either restriction enzyme digest [12] or denaturing gradient gel electrophoresis [13]. With these approaches, taxa detected in the gut microbiota of *Centruroides limpidus* and *Vaejovis smithi* included a predominance of Proteobacteria, in addition to Firmicutes, Actinobacteria and Spirochaetes [12]. In contrast, in *Androctonus australis* the gut and gonads included Firmicutes, Proteobacteria, Actinobacteria and Cyanobacteria, with Mollicutes being the most prevalent at 95% of all clones [13].

Novel lineages of Mollicutes have been described from gut tissue of *C*. *limpidus* and *V*. *smithi* [12]. One lineage termed Scorpion Group 1 (SG1) was found in *V*. *smithi*, but absent in *C*. *limpidus*, and had a 79% identity to *Spiroplasma lampyridicola*. Two other lineages, one in *V*. *smithi*, the other in *C*. *limpidus*, were M*ycoplasma*-related, being 88% identical to *Mycoplasma hyorhinis*. These 2 lineages together form a clade termed the Scorpion *Mycoplasma* Clade (SMC) [12]. Phylotypes corresponding to SMC have also been detected in *A*. *australis* [13].

A more detailed analysis using lineage-specific PCR primers and Sanger sequencing tested 23 scorpion morphospecies for the SG1 and SMC lineages [14]. The SMC lineage was detected in samples from both the Vaejovidae and Buthidae families, including *V*. *smithi* and *C*. *limpidus*, confirming their previous results [12]. SG1 was only detected in Vaejovidae, including additional *Vaejovis* and *Mesomexovis* species.

Beyond the SG1 lineage, additional *Spiroplasma*-like phylotypes have been detected in *C*. *limpidus*, *A*. *australis* and *Diplocentrus duende* scorpions. *Centruroides limpidus* contained a sequence 88% identical to *Spiroplasma platyhelix* that formed a clade with a sequence from a vinegaroon, *Mastigoproctus* sp. [14]. *Spiroplasma* sequences found in *A*. *australis* and *D*. *duende* were similar and grouped with a Citri-Chrysopicola-Mirum clade, thought to be derived from insect endosymbionts [14]. Whether *Mycoplasma-* and *Spiroplasma*-like phylotypes occur in other scorpion species is unknown.

The venom of venomous organisms has long intrigued researchers regarding its potential for clinically relevant natural products [15]. Venom-microbiomics is a newly emerging field that seeks to study venom-associated microbes found in a variety of different organisms, including snakes, spiders, insects, fish, *etc* [16]. Given that in some organisms venom toxicity can be enhanced by endogenous bacteria, that scorpion stings can result in bacterial infections [17], and scorpion venom contains antimicrobial compounds that can be produced by bacteria [18–21], it seems plausible that the venom-producing appendage, the telson, would harbor bacteria. To date there are few reports of bacteria in the telson. Attempts to grow bacteria from the venom of *H*. *arizonensis* and *Heterometrus spinifer* were inconsistent due in part to the variable and small volumes of venom isolated, despite having success culturing bacteria from the venom of numerous snake species [22, 23]. *Wolbachia* was detected in 10/20 (50%) of venom gland samples of the highly cytotoxic *Hemiscorpius lepturus* [9], however the study did not assess taxa beyond this single genus. In a separate report, the telson from the Old World buthid *A*. *australis*, a dangerously neurotoxic species, documented representatives belonging to Firmicutes, Actinobacteria, Proteobacteria and Flavobacteria taxa [13], although the sample size was limited to 3 animals and 7 bacterial isolates in total [13]. None of the telson samples represented bacterial class Mollicutes.

In this study we characterized the microbial diversity in telsons from 2 New World scorpion species representing different families, *Smeringurus mesaensis* of Vaejovidae and *Hadrurus arizonensis* of Hadruridae [24]. We used polymerase chain reaction (PCR) to create libraries of 16S rRNA gene sequences, commonly used in microbial barcoding, followed by sequencing to allocate taxonomic assignments. Phylogenetic analysis shows a rich diversity of bacteria in scorpion telsons, that are mostly species-specific, and have representatives from previously identified and novel Mollicutes phylotypes.

## Materials and methods

### Scorpion collection and dissection

**Fig 1:**
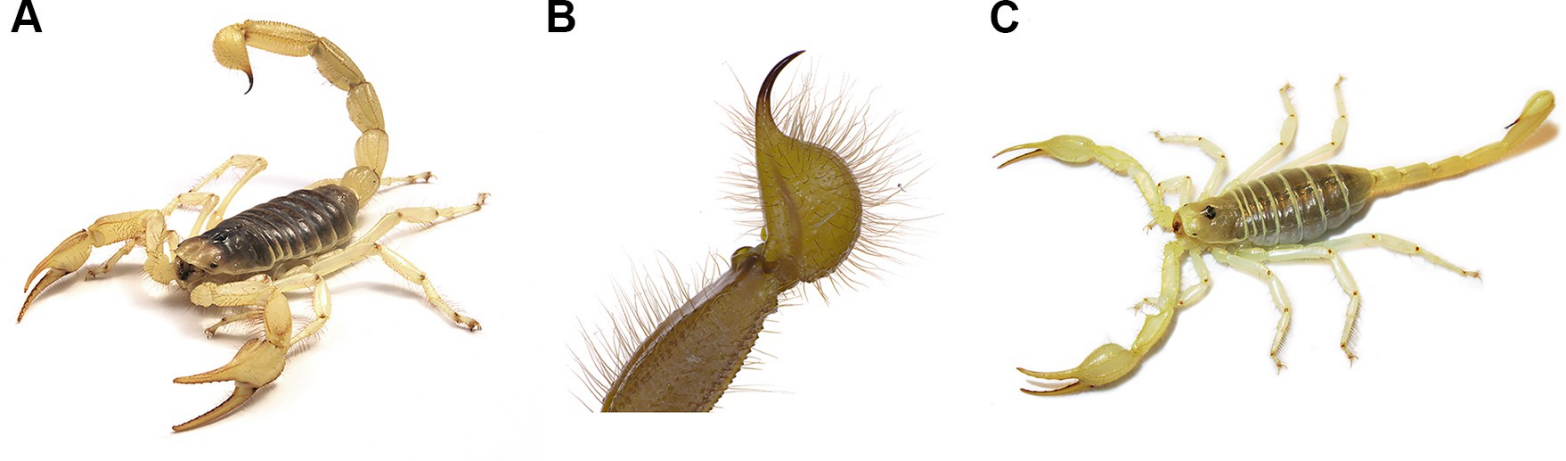
Images of *H*. *arizonensis* and *S*. *mesaensis*. Images of A, B) *H*. *arizonensis* with an enlarged view of its telson and stinger, and C) *S*. *mesaensis*.

We collected *H*. *arizonenesis* (Fig 1A and 1B; n = 7) from just east of Cattail Cove State Park, AZ (34.356159°, -114.147638°) and *S*. *mesaensis* (Fig 1C; n = 4) from Borrego Springs, CA (33.280283°, -116.292855°). For arachnid-related studies such as this, no ethics approval is required. The scorpions were held in captivity for 3–5 weeks without food or water, prior to freezing at -80°C until needed. During their captivity, each scorpion was housed individually within their own plastic container, to minimize contamination between scorpions. The scorpion exoskeleton was washed with 70% ethanol followed by water two times [12], prior to isolating telsons for DNA extraction. Under a dissecting microscope using sterile dissection tools, the telson was cut away from the rest of the metasoma and the aculeus was removed. The telson was subsequently bisected longitudinally to split it open, thus exposing the tissue inside. The dissection tools were sprayed with 70% ethanol and air-dried, prior to removal of the telson tissue. Sample tissue isolated for DNA extraction was referred to as venom gland or telson tissue, but could include the venom and venom gland tissue, plus the surrounding tissue, and the hemolymph. For each telson, the tissues were transferred to sterile 1.5 ml Eppendorf tubes in preparation for DNA extraction.

### DNA extraction from scorpion telsons

A modified Epicentre Master Pure DNA Purification (Lucigen) protocol was used to extract DNA from the scorpion telsons. Tissues were cut into small pieces, placed on ice and each sample was mixed with 300 μl Tissue and Cell Lysis Buffer and 50 μg Proteinase K. Samples were homogenized using a sterile cell homogenizer until a liquid suspension was made. Samples were incubated overnight in a 56°C shaking water bath. Each sample was iced and mixed vigorously with 175 μl of MPC Protein Precipitation Reagent and then centrifuged at 10,000 x g for 10 minutes at 4°C. Supernatants were mixed with 500 μl of cold -20°C isopropanol and stored at -20°C for 30 minutes to overnight. Samples were warmed to 4°C and centrifuged at 10,000 x g for 10 minutes at 4°C to pellet the DNA. Supernatants were decanted, and the pellets were washed twice with 70% ethanol. The pellets were air dried for 30 minutes and resuspended in 50 μL of TE buffer (10 mM Tris, 1 mM EDTA, pH 8).

### Amplification of 16S rRNA gene from scorpion telsons

Isolated DNA samples were subjected to PCR to test for the presence of bacteria in scorpion telsons using the 63F/1387R primers that specifically amplify the bacterial 16S rRNA gene from complex mixtures [25]. PCR mixtures of 25 μl contained the following: 50 to 200 ng of DNA template, 0.5 μM of each primer, 200 μM dNTPs, 2.5 units of Taq DNA Polymerase, 20 mM Tris and 50 mM KCl buffer, and 1.5 mM MgCl. The reaction conditions were 96°C for five minutes, 30 cycles at 94°C for 60 seconds, 58°C for 60 seconds, 72°C for one minute and 50 seconds, and a subsequent final extension at 72°C for 10 minutes. Electrophoresis of the PCR products (~1.4 kb 16S rRNA gene) was performed on a 1% (w/v) agarose gel in TAE with SYBR-Safe (1:10,000 Invitrogen). Gels were visualized with GelDoc (BioRad) software. Fresh PCR amplicons were used for TOPO cloning.

### TOPO TA cloning and transformation

TOPO TA cloning acts to separate the collection of 16S rRNA sequences found within the PCR amplicons into individual sequences by subcloning the sequences into plasmid vectors and growing the plasmids in bacteria [12]. DNA extracted from individual bacterial colonies serves as the template for a second vector-based PCR prior to Sanger sequencing, as described below. TOPO TA cloning (Invitrogen) was performed according to the manufacturer’s suggestions. Briefly, the reagents below were added to a tube in the order shown: 1 μL of fresh PCR product, 1 μL salt solution, 3 μL of water, and 1 μL of Invitrogen pCR^®^ 4-TOPO^®^ cloning plasmid. The reaction was mixed and incubated at room temperature for 30 minutes. The reactions were iced or stored at -20°C prior to transformations. The transformations occurred on ice, where 2 μL of the TOPO subcloning reaction was added to 50 μL of Transform One Shot competent cells (Invitrogen; hereafter termed competent cells). After 30 minutes, the competent cells were heat shocked for 30 seconds at 42°C, and returned to ice for 5 minutes. The competent cells were mixed with 250 μL of SOC medium (2% [w/v] Tryptone, 0.5% [w/v] Yeast Extract, 10 mM NaCl, 2.5 mM KCl, 10 mM MgCl2, 10 mM MgSO4, and 20 mM glucose) and incubated for 1 hour at 37°C in a shaking water bath (200 rpm). Fifty μL of cultured transformants were plated on LB agar with ampicillin (1% [w/v] bacto-tryptone, 0.5% [w/v] bacto-yeast extract, 1% [w/v] NaCl, 1.5% [w/v] agar, and 50 μg/mL ampicillin) and grown overnight at 37°C. Ampicillin was used to select for transformants containing plasmids with inserts. Negative controls included either competent cells alone without plasmids, or with plasmids lacking the addition of inserts. Colony growth was not detected on any of the negative controls.

### DNA isolation and PCR of transformant DNA

Individual transformants were subcultured in liquid broth with 50 μg/ mL ampicillin for 24 hours in a shaking water bath at 37°C. The cells were pelleted for 2 minutes at 8,000 x g and DNA isolated using a DNeasy (Qiagen) protocol, according to the manufacturer’s suggestions. DNA was eluted from the column using 100 μl of Buffer AE. The isolated DNA samples were subjected to PCR using M13 forward and reverse primers, whose binding sites are specific to the TOPO cloning vector, rather than to bacterial gene sequences. PCR mixtures of 25 μl contained the following: 1 μl DNA template, 0.5 μM of each primer, 200 μM dNTPs, 2.5 units of Taq DNA Polymerase, 20 mM Tris and 50 mM KCl buffer, and 1.5 mM MgCl. The reaction conditions were 96°C for 5 minutes, 30 cycles at 94°C for 30 seconds, 55°C for 30 seconds, 72°C for 1 minute, with a final extension at 72°C for 10 minutes. Electrophoresis of PCR products (~1.4 kb 16S rRNA gene) was performed on a 1% (w/v) agarose gel in TAE with SYBR-Safe (1:10,000 Invitrogen). Gels were visualized with GelDoc (BioRad) software. Positive controls included transformants with a known ~1.4 kb insert, whereas negative controls lacked template DNA.

### Analysis of sequencing results

PCR samples positive for the correctly sized insert of ~ 1.4 kb were cleaned with ExoSAP-IT (GE Healthcare, Piscataway, NJ, USA) prior to sequencing. PCR reactions (6 μl) were mixed with 2 μl ExoSAP-IT, incubated at 37°C and subsequently 80°C, each for 15 minutes. For sequencing, 3 μl of the ExoSAP-IT reaction was combined with 9 μl of PCR water per sample well. This yielded at least 70 ng/ μl of template per well. Primers were arrayed at ~3 pmol/ μl. Samples were shipped overnight at ambient temperature to the DNA Resource Core of Dana-Farber/Harvard Cancer Center; this core was funded in part by an NCI Cancer Center support grant 2P30CA006516-48. Sequencing reactions were performed on an ABI3730xl DNA analyzer. Each sample was sequenced in the forward direction. Ambiguous nucleotides were trimmed by hand in Geneious v.7.1.9 (Biomatters Ltd, Auckland, New Zealand). Chimeras were removed and the sequences were further trimmed upon submission to the National Center for Biotechnology Information (NCBI) Genbank under accession numbers OP050008—OP050060; OP050280—OP050295; OP050295—OP050317; OP047949—OP047976; OP050061—OP050062; OP050318—OP050319. The resulting sequences were used to identify bacteria using the NCBI Basic Local Alignment Search Tool (BLAST) [26]. Sequences were clustered into operational taxonomic units (OTUs) in mothur v.1.40.5 at a 3% cutoff with OptiClust [27], using SILVA (Silva seed v132) as a reference database.

### Phylogenetic analysis

To explore relationships among bacterial taxa, we generated two phylogenetic trees: 1) using all 16S sequences generated in this study, and 2) with samples from this study identified as belonging to class Mollicutes combined with representative Mollicutes sequences from the literature [14], the Ribosomal Database Project (RDP) [28], the Silva Database [29], and GenBank. For both analyses, sequences were aligned according to secondary structure using SSU-ALIGN v0.1.1 [30]. Using the program, we masked columns in which nucleotides were assigned with low confidence (S1 Fig). The alignments were used to generate Maximum Likelihood (ML) phylogenies with iQtree version 1.6.6 [31], implementing ModelFinder [32] to determine best-fit substitution models, and ultrafast bootstrap resampling to gauge nodal support [33]. The consensus phylogenies were visualized in FigTree v. 1.4.4 (http://tree.bio.ed.ac.uk/software/) and annotated in Adobe Illustrator.

### Diversity and statistical analysis

Based on the OTUs, alpha diversity was estimated as richness, evenness, and the Shannon index. A two-sampled t-test assuming unequal variance was used to compare mean values ± standard error, between the scorpion species. Beta diversity was measured using Bray-Curtis similarity and analyzed with permutational multivariate analysis of variance (PERMANOVA) and permutational multivariate analysis of dispersion (PERMDISP) with 2,000 permutations in Primer-E version 6 [34].

## Results

### The telson contains diverse bacterial taxa

To investigate the microbial diversity of the scorpion venom-producing appendage, the telson, we created 11, 16S rRNA libraries, 1 per telson (*S*. *mesaensis* n = 4; *H*. *arizonensis* n = 7). From a total of 267 clones, 40% (107 clones) yielded full-length products and were sent for sequencing. Some sequences contained chimeras that were split apart manually, thus yielding 114 sequences that were classified using NCBI Genbank.

**Fig 2:**
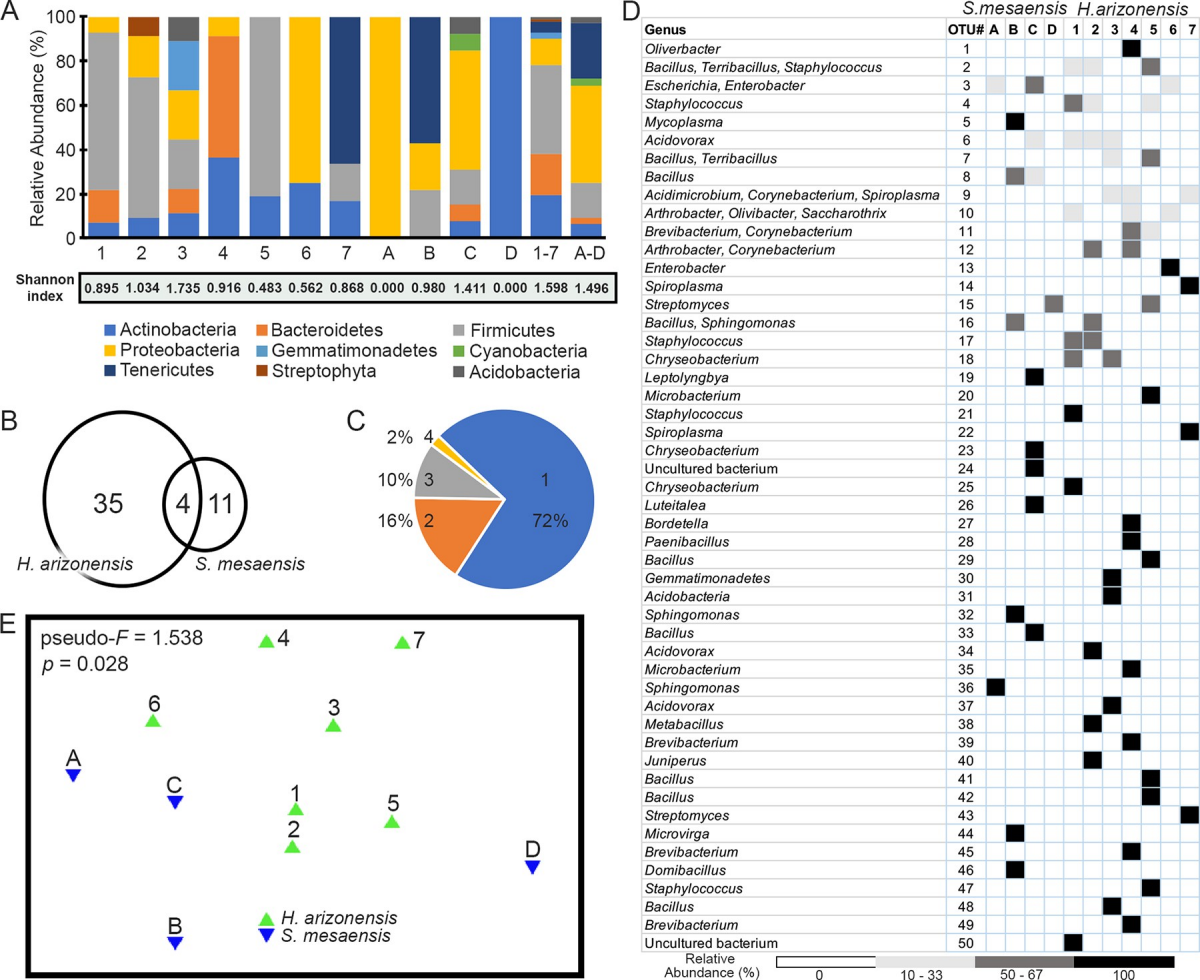
Composition of the *S*. *mesaensis* and *H*. *arizonensis* telson microbiota. A) Taxonomic assignments at the phylum level for individual samples and the aggregated data (right side) for each species. The Shannon index value for each sample is shown below the graph. B) The OTU distribution by species shows that most OTUs (46 out of 50) were unique to the host species; only 4 OTUs were common to both. C) The percentage distribution of OTUs shows the vast majority of OTUs were found in only one scorpion, and a single OTU was never found in more than four scorpions. D) Heatmap of the relative abundance (%) of each OTU and their assigned genera from BLAST results. The columns represent individual scorpions. The relative abundance is calculated as the percentage of OTU sequences per scorpion. White = 0%; light gray = 10–33%; dark gray = 50–67%; black = 100%. E) Bray-Curtis similarity and PERMANOVA indicate the centroids differ statistically between the microbiota in each scorpion species (pseudo-*F* = 1.538, *p* = 0.028, df = 10). Triangles represent individual scorpions; green–*H*. *arizonensis*, blue–*S*. *mesaensis*. For (A), (D), (E), 1 to 7 are *H*. *arizonensis*, A to D are *S*. *mesaensis*.

BLAST searches of sequenced clones revealed distinct differences between bacterial taxa found in *S*. *mesaensis* and *H*. *arizonensis*, indicating that each scorpion species has its own unique group of bacteria. Seven and 8 different phyla were detected in *S*. *mesaensis* and *H*. *arizonensis*, respectively. The most abundant phyla for *S*. *mesaensis* were Proteobacteria (44%), Tenericutes (25%) and Firmicutes (16%), whereas the most abundant phyla for *H*. *arizonensis* were Firmicutes (40%), Actinobacteria (20%), Bacteroidetes (18%), and Proteobacteria (12%; Fig 2A). Operational taxonomic unit (OTU) analysis showed that most OTUs clustered by host species, with 35 and 11 unique to *H*. *arizonensis* and *S*. *mesaensis*, respectively. Only 4 of the 50 (8%) OTUs were shared by both species (Fig 2B). The percentage distribution of OTUs showed that a single OTU could be found in 2 to 4 scorpions, representing 2% to 16% of all OTUs, but most OTUs were found in only 1 scorpion (72%) (Fig 2C and 2D). Based on the OTUs, measures of alpha diversity between the scorpion species using the mean Shannon indices were not statistically different (*H*. *arizonensis* 1.77 +/- 0.08, *S*. *mesaensis* 0.91 +/- 0.20, *p* = 0.11). Similarly, mean measures of richness (7.71 +/- 0.57, 4.25 +/- 0.83, *p* = 0.14) and evenness (0.92 +/- 0.02, 0.60 +/- 0.10, *p* = 0.22) for *H*. *arizonensis* and *S*. *mesaensis* respectively, showed no significant differences. The ordination plot of beta diversity based on Bray-Curtis similarity (Fig 2E) showed the centroids differed between the scorpion species (pseudo-*F* = 1.538, df = 10, *p* = 0.028), but their dispersion did not (*F* = 0.381, *p* = 0.651). These data indicate a significant difference in diversity between the microbiota of *H*. *arizonensis* and *S*. *mesaensis*, but not within the respective species.

### The 16S rRNA sequences from the cloned telson libraries are species-specific

**Fig 3:**
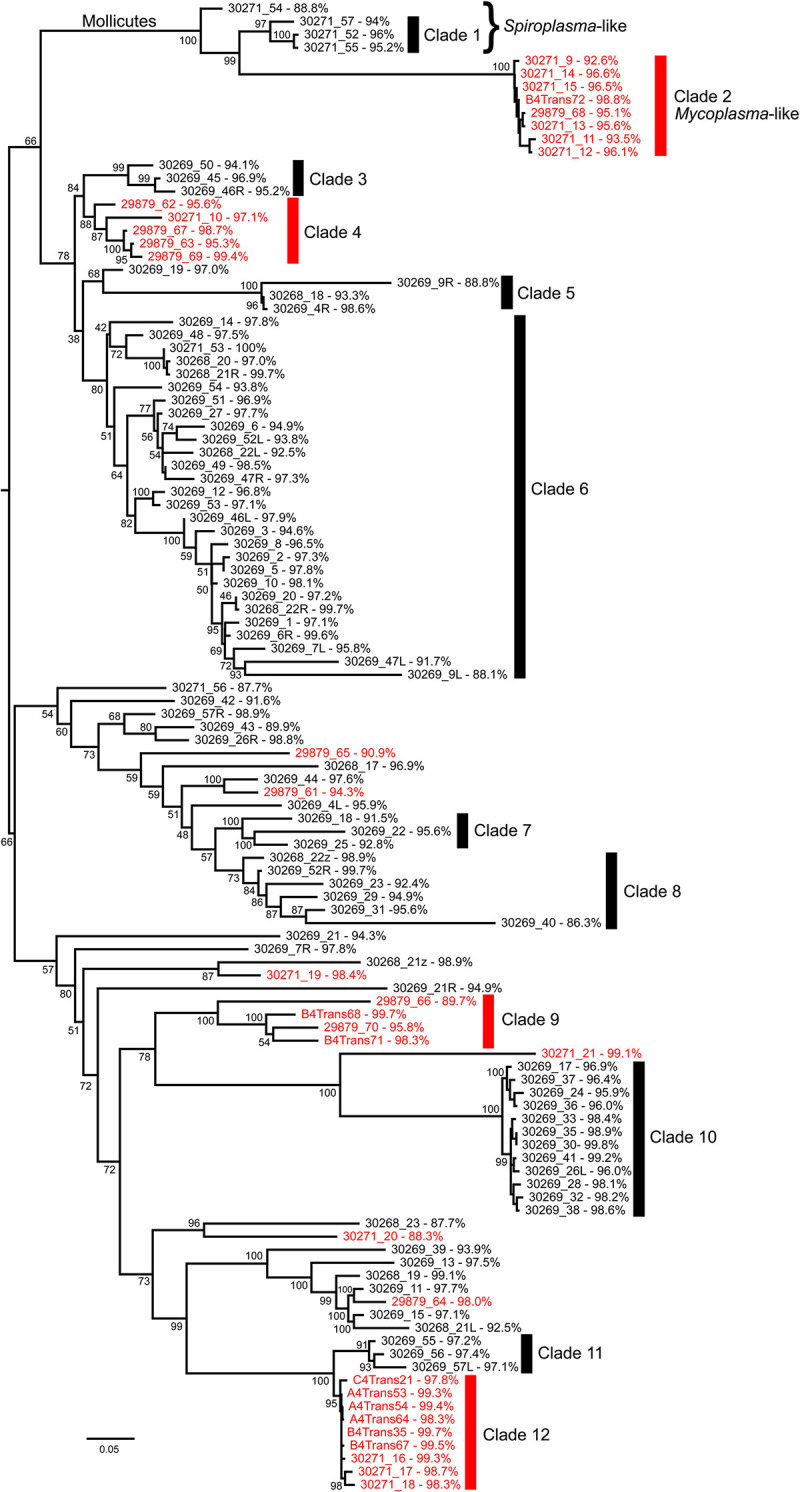
Phylogenetic tree showing species-specific clades for telson bacteria isolated from *S*. *mesaensis* and *H*. *arizonensis*. A Maximum Likelihood phylogeny was constructed based on the DNA sequences of 114 16S rRNA samples isolated from *S*. *mesaensis (n = 32)* and *H*. *arizonensis (n = 82)* telsons. Scorpion-specific clades are identified by vertical bars numbered 1–12 with samples from *H*. *arizonensis* indicated in black; *S*. *mesaensis* indicated in red. We considered clades to be scorpion-specific if they contained 3 or more taxa from a single scorpion species and were supported by bootstrap values greater than 70.

BLAST searches returned identity scores ranging from 86.3% to 100% (S1 Table). Scorpions of the same species shared many common bacterial genera. For example, 50% of *S*. *mesaensis* had *Escherichia*, *Sphingomonas* or *Bacillus*, whereas 71% of *H*. *arizonensis* had *Bacillus* and 43% had *Staphlococcus* (S1 Table). Interestingly, Mollicutes phylotypes were detected in both scorpion species. *Mycoplasma* was detected in 8 clones from 1 *S*. *mesaensis* scorpion whose identities were most like uncultured bacterial clones from the gut tissues of *V*. *smithi* and *Mesomexovis* aff. *punctatus* [12] and clustered into clade 2 (Fig 3). One *H*. *arizonensis* specimen contained 4 clones of *Spiroplasma* with identities from 88.8 to 95.8% akin to *Spiroplasma platyhelix* (S1 Table). These *Spiroplasma*-like sequences grouped into clade 1 (Fig 3).

The highly structured topology seen in Fig 3 suggests considerable genetic diversity among microbes from scorpion telsons. The tree shows 4 clades of exclusively *S*. *mesaensis* and 8 clades exclusively of *H*. *arizonensis* samples. Only 5 samples from *S*. *mesaensis* nested within *H*. *arizonensis* samples. No *H*. *arizonensis* samples nested within the *S*. *mesaensis* groups. These data indicate that most sequences cluster into clades that are species-specific. Samples with low values for percent identity tend to form long branches, suggesting the presence of unique microbes in these scorpion species (Fig 3).

### 16S rRNA phylogeny shows sequence contributions to the scorpion *Mycoplasma* clade and a novel *Spiroplasma*-like lineage

**Fig 4:**
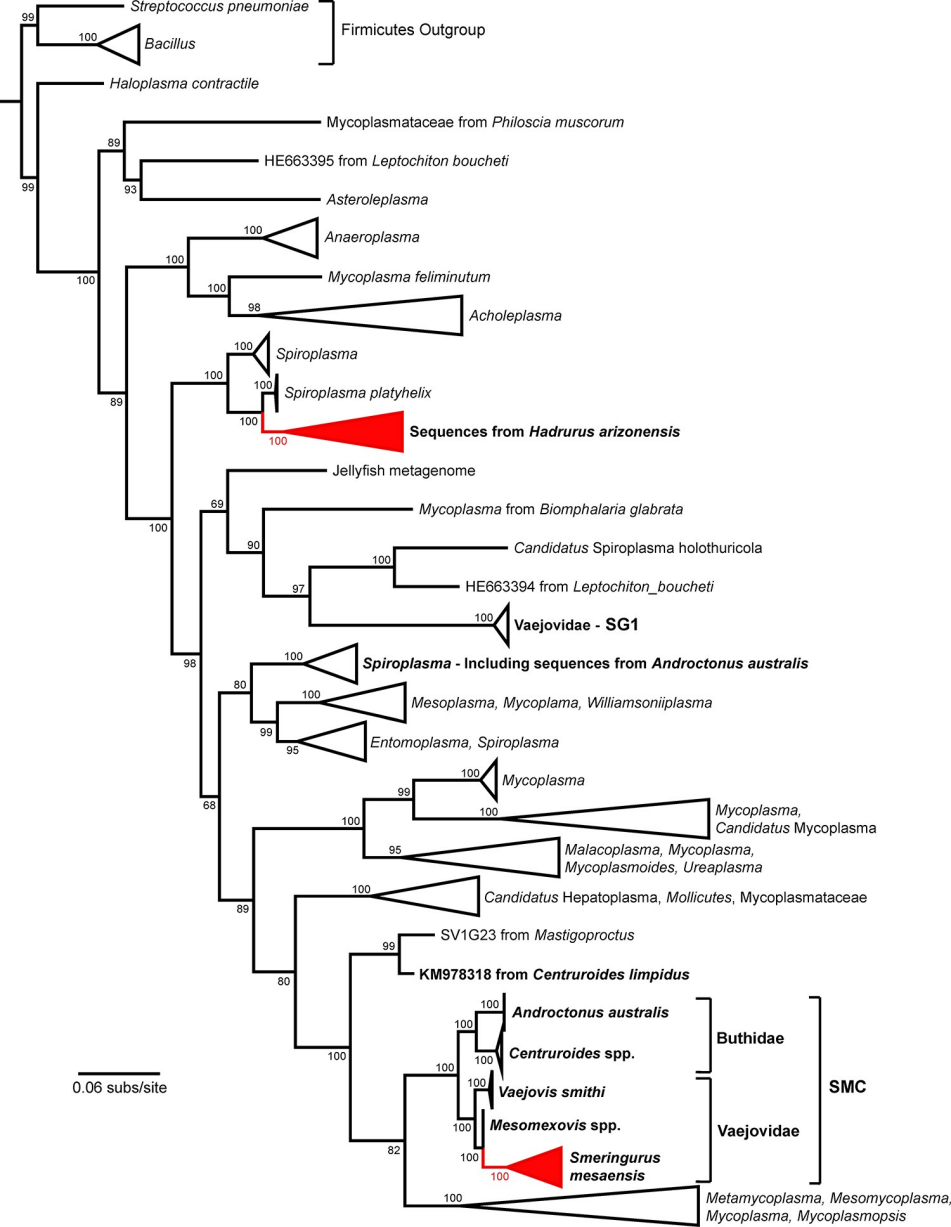
Phylogenetic tree of Mollicutes 16S rRNA. The phylogenetic tree was constructed using Maximum Likelihood, including a total of 312 sequences representing Mollicutes taxa. Scorpion sequences are in bold. Red collapsed regions indicate 12 sequences reported in this study from *H*. *arizonensis* and *S*. *mesaensis*. For an expanded version of this figure see S2 Fig.

To determine the relationship between our 16S rRNA *Mycoplasma*-like and *Spiroplasma*-like sequences to Mollicutes, we sourced sequences from public databases and the literature (312 in total) to create a phylogenetic tree (Fig 4 and S2 Fig). The *Mycoplasma*-like sequences from *S*. *mesaensis* were grouped as part of the previously designated Scorpion *Mycoplasma* Clade [12, 14], that includes scorpions from both the Buthidae and Vaejovidae families. Within this clade, the *S*. *mesaensis* sequences formed a tightly grouped sister clade with other Vaejovidae family members, being more closely related to sequences from *Mesomexovis spp*. compared to *V*. *smithi* (Fig 4). The node values (100) indicate that these relationships are highly supported. The *Spiroplasma*-like sequences from *H*. *arizonensis* grouped with other *Spiroplasma* sequences, forming a sister clade with sequences from *Spiroplasma platyhelix* with high node values (100; Fig 4). These *H*. *arizonensis* sequences appear to form a novel lineage that is unrelated to previously reported scorpion sequences.

## Discussion

Given the importance of the microbiome to the success of several organisms [3–5], and indirect evidence suggesting the presence of bacteria associated with the scorpion venom and venom organ [18–21], we tested whether the venom organ of *H*. *arizonensis* and *S*. *mesaensis* harbored bacteria. To the best of our knowledge, we have provided the first evidence of the broad bacterial diversity in the telsons of *H*. *arizonensis* and *S*. *mesaensis*. Interestingly, each scorpion species has its own set of telson bacteria (Fig 2) that group by host species in phylogenetic analyses (Figs 3 and 4). Sequences from *S*. *mesaensis* added another Vaejovidae family member to the previously reported Scorpion *Mycoplasma* Clade and a novel lineage of *Spiroplasma*-like sequences was found in *H*. *arizonensis* (Fig 4). Our study contributes to the emerging field of venom-microbiomics that seeks to integrate the areas of venomics with microbiology [16].

In this study we housed the scorpions in individual containers prior to tissue isolation to reduce the chance of cross contamination between animals and isolated the telson/venom gland samples under sterile conditions with a dissecting microscope. Despite our best efforts to mitigate potential sources of contamination, we cannot rule out that regions beyond the venom gland itself, like the hemolymph or exoskeleton, may have contributed microbiota.

Previous research has shown that the scorpion gut microbiome is largely species-specific [12, 14]. Similarly, our data indicate distinct telson microbiomes in *S*. *mesaensis* compared to *H*. *arizonensis* (Figs 2–4). At the phylum level, we found each scorpion species to have its own set of bacteria (Fig 2). Given that Proteobacteria make up the largest bacterial phylum, it is not surprising that these Gram-negative bacteria were predominant in *S*. *mesaensis* (Fig 2). A predominance of Proteobacteria is consistent with previous studies in *V*. *smithi* and *C*. *limpidus* [12], but contrary to the findings in *A*. *australis*, where 95% of the clones from gut and gonad tissues were Mollicutes [13]. Unlike *S*. *mesaensis*, Proteobacteria were limited in abundance in *H*. *arizonensis*, where Firmicutes dominated (Fig 2). Only 8% of OTUs were shared between the species and the Bray-Curtis similarity showed that the centroids but not the dispersion were significantly different (Fig 2), providing additional and statistical evidence for specificity of the microbiomes by species.

Our phylogenetic tree from the cloned libraries showed that the sequences separate into 12 scorpion-specific clades, with 4 clades from *S*. *mesaensis* and 8 clades from *H*. *arizonensis* (Fig 3). Of the 114 sequences analyzed, 5 *S*. *mesaensis* sequences were nested within *H*. *arizonensis* samples; none of *H*. *arizonensis* samples nested within groups of *S*. *mesaensis* sequences. Albeit the prevailing trend showed clustering of the bacterial 16S rRNA sequences, according to their host species.

As aforementioned, the 114 bacterial clones isolated from scorpion telsons represented a broad range of taxa including some which are related to *Mycoplasma* and *Spiroplasma*. *Mycoplasma* and *Spiroplasma* are from the Mollicutes class of bacteria, and their effects on hosts range from pathogenic, to commensal, to beneficial [35]. Mollicutes hallmarks are a lack of a cell wall and extremely small size, often having widths of less than 0.2 μm. They can reside on the outer surface of cells, inside of cells or extracellularly. *Spiroplasma* are typically found in plants and arthropods, as are *Mycoplasma*, that are additionally found in vertebrate animals. *Mycoplasma* and *Spiroplasma* were of particular interest since in other scorpion species, these genera formed novel lineages that were related to gut microbiota [12, 14], and Mollicutes phylotypes were predominant in the tissues of *A*. *australis* [13]. For *S*. *mesaensis*, from a single scorpion we retrieved 8 *Mycoplasma*-like clones that formed clade 2 (Fig 3). Sequences from this clade showed close identity with sequences from various scorpion species including for example, *V*. *smithi* (94%; KM978265.2), *M*. aff. *punctatus* (97%; MF134711 and MF134712), *C*. *limpidus* (91%; KM978292.1) [14], and *A*. *australis* (91%; KT880638.1) [13]. *Mycoplasma* had previously been detected in 2 scorpion families, Vaejovidae and Buthidae [12–14], forming a novel clade termed the Scorpion *Mycoplasma* Clade, with separate branches for each family [12, 14]. Our results provide an additional Vaejovidae family member to this small albeit ever-expanding Scorpion *Mycoplasma* Clade, *S*. *mesaensis*, that is most closely related to *Mycoplasma* found in *Mesomexovis* species (Fig 4). This result provides additional evidence of cospeciation between scorpions and Scorpion *Mycoplasma* Clade symbionts, as previously proposed [14]. *Mycoplasma* has not yet been detected in scorpions beyond these 2 families.

From a single *H*. *arizonensis* scorpion we detected 4 sequences most closely related to *Spiroplasma platyhelix* (Fig 3 and S1 Table) [36]. The ML analysis grouped all 4 sequences into a single clade that included *Spiroplasma platyhelix*, and formed a sister clade with *Spiroplasma* sequences from other arthropods, including beetles and ticks (Fig 4 and S2 Fig) [37, 38]. Node values (100) highly support this topology.

The detection of *Spiroplasma* in discrete scorpion families and species is increasing. Including this study, endogenous *Spiroplasma* sequences have been detected in 3 scorpion families and 8 species as follows: 1. Hadruridae—*H*. *arizonensis* (this study); 2. Vaejovidae*–V*. *smithi*, *Vaejovis granulatus*, *M*. aff. *punctatus*, *Mesomexovis* aff. *oaxaca*, *Mesomexovis* aff. *variegatus;* 3. Buthidae*–C*. *limpidus*, *A*. *australis*. [12–14]. Not included in this tally are *Spiroplasma* sequences from *D*. *duende* and *A*. *australis*, that were likely derived from endosymbionts of insect prey, rather than forming part of the endogenous microbiome of the scorpions themselves [13, 14].

*Spiroplasma*-like sequences of the Vaejovidae family includes the novel lineage termed Scorpion Group 1 (SG1) which are thus far restricted to this family alone [12, 14]. Our phylogenetic analysis indicates that SG1 represents a distinct lineage with low haplotype diversity that is most closely related to sequences from the mollusk *Leptochiton boucheti* (Fig 4). This latter prediction has strong node support (bootstrap (bs) = 97) and differs from previous reports [12, 14], likely due to differing analytical methods. *Spiroplasma* taxa from the Buthidae family are divergent, with the *A*. *australis* sequences grouped into a clade combined with several other *Spiroplasma* species, whereas OTU4 from *C*. *limpidus* formed a sister clade with samples from a vinegaroon, *Mastigoproctus sp*., as it did previously [14]. The predicted phylogenetic relationship between *C*. *limpidus* and *Mastigoproctus* is strongly supported (bs = 99). The *Spiroplasma* detected in *H*. *arizonensis* are divergent from the sequences of OTU4 (85% identity), SG1 (76–77% identity or unrelated) and *A*. *australis* (84–86% identity), and thus form a novel scorpion lineage (Fig 4).

Although our sequence data from *S*. *mesaensis* and *H*. *arizonensis* provide great insights into telson microbiomes, the actual bacterial diversity is likely to be much greater. TOPO cloning allowed us to accurately identify several bacterial phylotypes, but high throughput sequencing methods would provide a more detailed data set. Additionally, our analyses are constrained by the quality and number of sequences in the reference database and we acknowledge that the 16S rRNA gene sequence alone is not sufficient to assign all taxa to species. Notwithstanding the limitations, our survey is the first to report bacteria in the telsons of *H*. *arizonenesis* (the first hadrurid) and *S*. *mesaensis*, complementing the detection of telson bacteria in *A*. *australis* [13] and *H*. *lepturus* [9]. Further, our results indicate that different scorpion species contain unique suites of microbes (Figs 2 and 3).

While the gut microbiome can be influenced by several factors including genetics, environment, and diet [12, 14, 39], which of these factors can alter the telson microbiome is unknown. Horizontal acquisition of bacterial symbionts is not likely since, for example, the sequences we isolated are not closely related to insect endosymbionts (S1 Table). However, there is evidence supporting the vertical transmission of bacterial symbionts in scorpions. Previous studies showed that in 2 species, *M*. aff. *punctatus* and *Centruroides noxius*, scorpion embryos and their mothers’ gut had identical Mollicutes bacterial symbionts [14], and bacteria have been detected in the gonads of *A*. *australis* [13]. Further, evidence suggests a coevolution between scorpion species and their *Mycoplasma* and *Spiroplasma* symbionts, SMC and SG1 taxa, dating back to a common ancestor from more than 120 million years ago [14]. More research is needed within and between scorpion species and scorpion families to determine the association of their bacterial symbionts.

What function might the telson microbiome provide? Bacteria in some organisms, like antlion larvae or pufferfish, function to produce or increase the toxicity of the venom, making the venom more effective [40, 41]. This would increase the chances of successful defense and prey capture. It may be that the telson microbiome also plays a role in the biochemistry and toxicity of the scorpion venom. An additional explanation is that the telson bacteria protect the scorpion from other microorganisms. For example, the genome of “*Candidatus*” Spiroplasma holothuricola, isolated from sea cucumbers in a deep-sea ecosystem, contained genes from the clustered regularly interspaced short palindromic repeats (CRISPR) /Cas system and toxins for defending against microbes [42]. Further, antimicrobial peptides may provide innate immunity. Several studies have detected antimicrobial peptides in scorpion venom, where they are assumed to be produced by the scorpion [43]. However, based on our results we propose that some of the antimicrobial peptides may be produced by the telson bacteria. One would expect that since the scorpion telson contains bacteria, the venom should also contain bacteria. Indeed, bacteria have been successfully isolated from the venom of snakes and spiders [23], even though venom extraction and analysis can be challenging. For example, Esmaeilishirazifard et al., 2018 [22], attempted to culture bacteria from scorpion venoms, including *H*. *arizonensis* studied here, but no bacterial growth was reported, due to sampling limitations that included scorpion availability and low volumes (<1 to 30 ul) of venom. Despite these challenges it would be interesting to re-explore scorpion venom for bacteria in the future.

## Conclusions

Here we provide the first molecular evidence of bacteria in the telsons of the scorpion species *S*. *mesaensis* and *H*. *arizonensis* and reveal unique microbiomes, including novel bacterial lineages, from the 2 species tested. Given the emerging importance of the microbiome for health and well-being in numerous organisms, it is not surprising that an ancient appendage involved in food acquisition and defense, has its own microbiome. Although the precise function of the telson microbiome is unknown, our findings may change how we view the biology of scorpions, their venoms, and the microscopic life that lives within them.

## Supporting information

S1 Fig Secondary structure of 16S rRNA showing sites that were included and excluded (masked) in phylogenetic analysis of Mollicutes phylotypes. The figure was created with the SSU-ALIGN package (http://eddylab.org/software.html) which derived the structure diagram from the CRW database (http://www.rna.ccbb.utexas.edu/).
(PDF)

S2 Fig Expanded phylogenetic tree of Mollicutes 16S rRNA. (PDF)

S1 Table Identification using DNA sequencing of the bacteria isolated from scorpions, their percent identity as indicated by BLAST, clade organization, and OTU assignment. (DOCX)
