# Supplementary figures and images for: A first molecular characterization of the scorpion telson microbiota of Hadrurus arizonensis and Smeringurus mesaensis
Source: PLoS One. 2023 Jan 17;18(1):e0277303. doi: 10.1371/journal.pone.0277303 (PMC9844838; doi:10.1371/journal.pone.0277303)

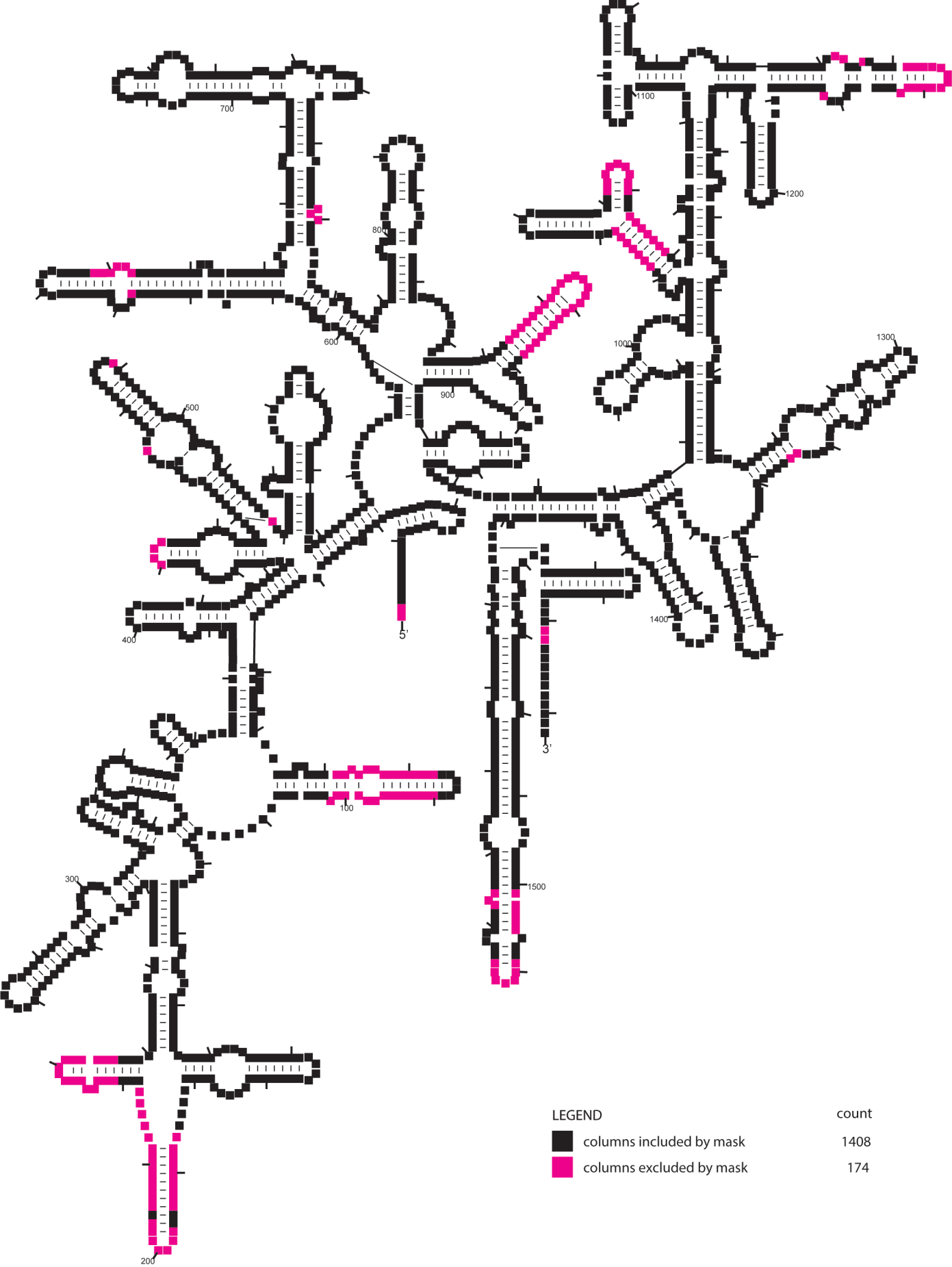

LEGEND

columns included by mask

columns excluded by mask

count

1408

174

Supplement: S1 Fig — The figure was created with the SSU-ALIGN package (http://eddylab.org/software.html) which derived the structure diagram from the CRW database (http://www.rna.ccbb.utexas.edu/). (PDF) [file pone.0277303.s001.pdf]

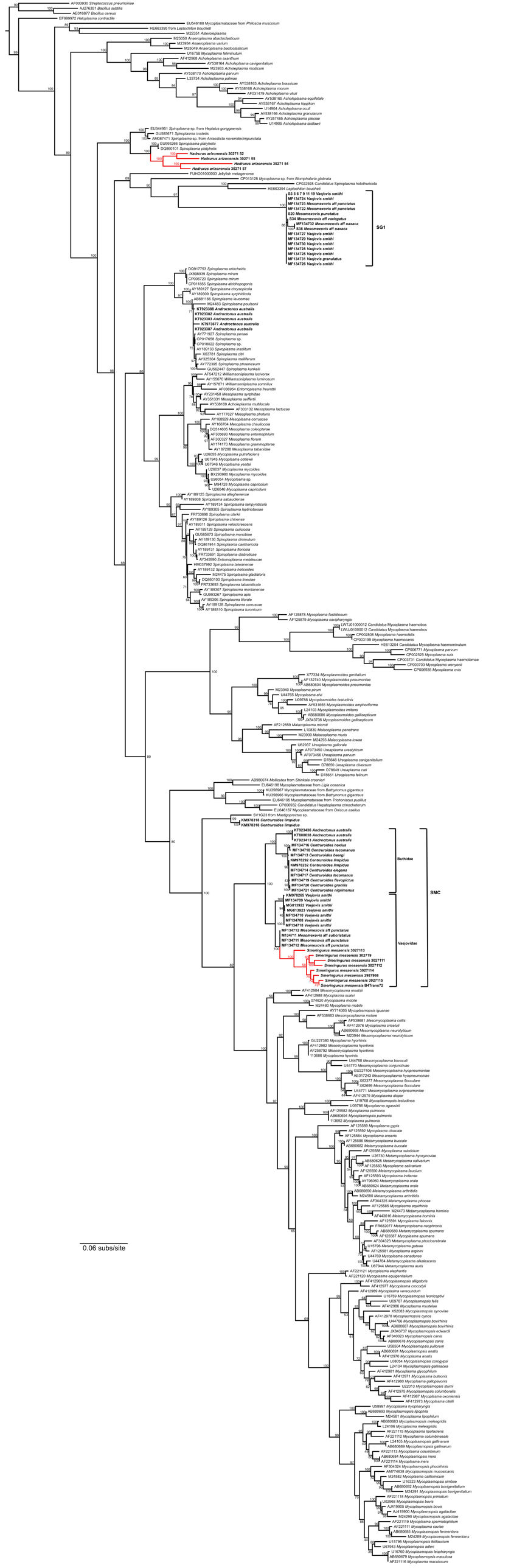

Supplement: S2 Fig — (PDF) [file pone.0277303.s002.pdf]
