# Supplementary material for: A first molecular characterization of the scorpion telson microbiota of Hadrurus arizonensis and Smeringurus mesaensis
Source: PLoS One. 2023 Jan 17;18(1):e0277303. doi: 10.1371/journal.pone.0277303 (PMC9844838; doi:10.1371/journal.pone.0277303)
Supplement: S1 Table — (DOCX) [file pone.0277303.s003.docx]

**S1 Table. Identification of bacterial sequences isolated from scorpion telsons, their percent identity as indicated by BLAST, and OTU assignment.**

| **OTU #** | **Sample ID** | **Scorpion Species** | **Scorpion**  **ID** | **Closest relative** | **% Identity** |
| --- | --- | --- | --- | --- | --- |
| 4 | 30269_10 | *H. arizonensis* | 1 | *Staphylococcus* sp. | 98.1 |
| 6 | 30269_11 | *H. arizonensis* | 1 | *Acidovorax* sp. | 97.7 |
| 4 | 30269_2 | *H. arizonensis* | 1 | *Staphylococcus* sp. | 97.3 |
| 2 | 30269_3 | *H. arizonensis* | 1 | *Staphylococcus* sp. | 94.6 |
| 10 | 30269_4L | *H. arizonensis* | 1 | *Saccharothrix* sp. | 95.9 |
| 18 | 30269_4R | *H. arizonensis* | 1 | *Chryseobacterium taihuense* | 98.6 |
| 4 | 30269_5 | *H. arizonensis* | 1 | *Staphylococcus* sp. | 97.8 |
| 2 | 30269_6L | *H. arizonensis* | 1 | *Bacillus cereus* | 94.9 |
| 17 | 30269_6R | *H. arizonensis* | 1 | *Staphylococcus* sp. | 99.6 |
| 4 | 30269_7L | *H. arizonensis* | 1 | *Staphylococcus kloosii* | 95.8 |
| 50 | 30269_7R | *H. arizonensis* | 1 | Uncultured *Firmicutes* bacterium | 97.8 |
| 4 | 30269_8 | *H. arizonensis* | 1 | *Staphylococcus* sp. | 96.5 |
| 21 | 30269_9L | *H. arizonensis* | 1 | *Staphylococcus gallinarum* | 88.1 |
| 25 | 30269_9R | *H. arizonensis* | 1 | *Chryseobacterium* sp. | 88.8 |
| 6 | 30268_19 | *H. arizonensis* | 2 | *Acidovorax wautersii* | 99.1 |
| 34 | 30268_21L | *H. arizonensis* | 2 | *Acidovorax* sp. | 89.8 |
| 16 | 30268_21R | *H. arizonensis* | 2 | *Bacillus subtilis* | 99.7 |
| 40 | 30268_21z | *H. arizonensis* | 2 | *Juniperus saltuaria* | 98.9 |
| 2 | 30268_22L | *H. arizonensis* | 2 | *Staphylococcus pseudintermedius* | 92.5 |
| 17 | 30268_22R | *H. arizonensis* | 2 | *Staphylococcus saprophyticus* | 99.7 |
| 12 | 30268_22z | *H. arizonensis* | 2 | *Arthrobacter* sp. | 100 |
| 4 | 30269_1 | *H. arizonensis* | 2 | *Staphylococcus* sp. | 97.1 |
| 2 | 30269_12 | *H. arizonensis* | 2 | *Bacillus anthracis* | 96.8 |
| 38 | 30269_19 | *H. arizonensis* | 2 | *Metabacillus* sp. | 97.0 |
| 4 | 30269_20 | *H. arizonensis* | 2 | *Staphylococcus gallinarum* | 97.2 |
| 9 | 30268_17 | *H. arizonensis* | 3 | Uncultured *Acidimicrobiales* | 94.3 |
| 18 | 30268_18 | *H. arizonensis* | 3 | *Chryseobacterium taihuense* | 93.3 |
| 7 | 30268_20 | *H. arizonensis* | 3 | *Bacillus subtilis* | 97.0 |
| 31 | 30268_23 | *H. arizonensis* | 3 | *Acidobacteria* | 87.7 |
| 37 | 30269_13 | *H. arizonensis* | 3 | *Acidovorax* sp. | 97.5 |
| 48 | 30269_14 | *H. arizonensis* | 3 | *Bacillus* sp. | 97.8 |
| 6 | 30269_15 | *H. arizonensis* | 3 | *Acidovorax* sp. | 97.1 |
| No  analysis | 30269_21L | *H. arizonensis* | 3 | Uncultured *Gemmatimonadetes* bacterial clone | 94.3 |
| 30 | 30269_21R | *H. arizonensis* | 3 | Uncultured *Gemmatimonadetes* bacterial clone | 94.9 |
| 1 | 30269_17 | *H. arizonensis* | 4 | *Olivibacter* sp. | 96.9 |
| 12 | 30269_18 | *H. arizonensis* | 4 | Uncultured bacterium *(*similar to *Corynebacterium variabile)* | 91.5 |
| 11 | 30269_22 | *H. arizonensis* | 4 | *Corynebacterium variabile* | 95.6 |
| 49 | 30269_23 | *H. arizonensis* | 4 | *Brevibacterium pigmentatum* | 92.4 |
| 1 | 30269_24 | *H. arizonensis* | 4 | *Olivibacter* sp. | 95.9 |
| 9 | 30269_25 | *H. arizonensis* | 4 | *Corynebacterium* sp. | 92.8 |
| 1 | 30269_26L | *H. arizonensis* | 4 | *Olivibacter* sp. | 96.0 |
| 35 | 30269_26R | *H. arizonensis* | 4 | *Microbacterium* sp. | 98.8 |
| 1 | 30269_28 | *H. arizonensis* | 4 | *Olivibacter* sp. | 98.1 |
| 45 | 30269_29 | *H. arizonensis* | 4 | *Brevibacterium* sp. | 94.9 |
| 1 | 30269_30 | *H. arizonensis* | 4 | *Olivibacter* sp. | 99.8 |
| 11 | 30269_31 | *H. arizonensis* | 4 | *Brevibacterium* sp. | 95.6 |
| 1 | 30269_32 | *H. arizonensis* | 4 | *Olivibacter* sp. | 98.2 |
| 1 | 30269_33 | *H. arizonensis* | 4 | *Olivibacter* sp. | 98.4 |
| 1 | 30269_35 | *H. arizonensis* | 4 | *Olivibacter* sp. | 98.9 |
| 1 | 30269_36 | *H. arizonensis* | 4 | *Olivibacter* sp. | 96.0 |
| 10 | 30269_37 | *H. arizonensis* | 4 | *Olivibacter* sp. | 96.4 |
| 1 | 30269_38 | *H. arizonensis* | 4 | *Olivibacter* sp. | 98.6 |
| 27 | 30269_39 | *H. arizonensis* | 4 | *Bordetella* sp. | 93.9 |
| 39 | 30269_40 | *H. arizonensis* | 4 | Uncultured *Brevibacterium* sp. | 86.3 |
| 1 | 30269_41 | *H. arizonensis* | 4 | *Olivibacter* sp. | 99.2 |
| 28 | 30269_42 | *H. arizonensis* | 4 | *Paenibacillus* sp. | 91.6 |
| 2 | 30269_27 | *H. arizonensis* | 5 | *Bacillus paramycoides* | 97.7 |
| 20 | 30269_43 | *H. arizonensis* | 5 | *Microbacterium foliorum* | 89.9 |
| 15 | 30269_44 | *H. arizonensis* | 5 | *Streptomyces* sp. | 97.6 |
| 7 | 30269_45 | *H. arizonensis* | 5 | *Terribacillus halophilus* | 96.9 |
| 4 | 30269_46L | *H. arizonensis* | 5 | *Staphylococcus succinis* | 97.9 |
| 7 | 30269_46R | *H. arizonensis* | 5 | *Terribacillus* sp. | 95.2 |
| 47 | 30269_47L | *H. arizonensis* | 5 | *Staphylococcus gallinarum* | 91.7 |
| 41 | 30269_47R | *H. arizonensis* | 5 | *Bacillus anthracis* | 97.3 |
| 42 | 30269_48 | *H. arizonensis* | 5 | *Bacillus cereus* | 97.5 |
| 2 | 30269_49 | *H. arizonensis* | 5 | *Bacillus cereus* | 98.5 |
| 2 | 30269_50 | *H. arizonensis* | 5 | *Terribacillus* sp. | 94.1 |
| 2 | 30269_51 | *H. arizonensis* | 5 | *Bacillus paramycoides* | 96.9 |
| 2 | 30269_52L | *H. arizonensis* | 5 | *Bacillus mycoides* | 93.8 |
| 11 | 30269_52R | *H. arizonensis* | 5 | *Brevibacterium linens* | 99.7 |
| 2 | 30269_53 | *H. arizonensis* | 5 | *Bacillus anthracis* | 97.1 |
| 29 | 30269_54 | *H. arizonensis* | 5 | *Bacillus* sp. | 93.8 |
| 13 | 30269_55 | *H. arizonensis* | 6 | *Enterobacter hormaechei* | 97.2 |
| 13 | 30269_56 | *H. arizonensis* | 6 | *Enterobacter* sp. | 97.4 |
| 3 | 30269_57L | *H. arizonensis* | 6 | *Enterobacter hormaechei hoffmannii* | 97.1 |
| 10 | 30269_57R | *H. arizonensis* | 6 | *Arthrobacter* sp. | 98.9 |
| 14 | 30271_52 | *H. arizonensis* | 7 | *Spiroplasma platyhelix* | 95.8 |
| No  analysis | 30271_53 | *H. arizonensis* | 7 | *Bacillus subtilis* | 100 |
| 9 | 30271_54 | *H. arizonensis* | 7 | *Spiroplasma platyhelix* | 88.8 |
| 14 | 30271_55 | *H. arizonensis* | 7 | *Spiroplasma platyhelix* | 95.2 |
| 43 | 30271_56 | *H. arizonensis* | 7 | *Streptomyces* sp. | 87.7 |
| 22 | 30271_57 | *H. arizonensis* | 7 | *Spiroplasma platyhelix* | 93.6 |
| 36 | 29879_70 | *S. mesaensis* | A | *Sphingomonas echinoides* | 95.8 |
| 3 | A4Trans53 | *S. mesaensis* | A | *Escherichia coli* | 99.3 |
| 3 | A4Trans54 | *S. mesaensis* | A | *Escherichia coli* | 99.4 |
| 3 | A4Trans64 | *S. mesaensis* | A | *Escherichia coli* | 98.3 |
| 44 | 29879_66 | *S. mesaensis* | B | *Microvirga subterranea* | 89.7 |
| 8 | 29879_67 | *S. mesaensis* | B | *Bacillus* sp. | 98.7 |
| 5 | 29879_68 | *S. mesaensis* | B | Uncultured bacterium clone M23 - *Mesomexovis* aff *punctatus* | 95.1 |
| 8 | 29879_69 | *S. mesaensis* | B | *Bacillus* sp. | 99.4 |
| 46 | 30271_10 | *S. mesaensis* | B | *Domibacillus robiginosus* | 97.1 |
| 5 | 30271_11 | *S. mesaensis* | B | Uncultured bacterium clone L4 8Scorpion *Mycoplasma* - *V. smithi* | 93.5 |
| 5 | 30271_12 | *S. mesaensis* | B | Uncultured bacterium clone L4 1Scorpion *Mycoplasma -V. smithi* | 96.1 |
| 5 | 30271_13 | *S. mesaensis* | B | Uncultured bacterium clone M23 - *Mesomexovis* aff *punctatus* | 95.6 |
| 5 | 30271_14 | *S. mesaensis* | B | Uncultured bacterium clone L4 8Scorpion *Mycoplasma - V. smithi* | 96.6 |
| 5 | 30271_15 | *S. mesaensis* | B | Uncultured bacterium clone L4 8Scorpion *Mycoplasma - V. smithi* | 96.8 |
| 5 | 30271_9 | *S. mesaensis* | B | Uncultured bacterium clone L4 8Scorpion *Mycoplasma - V. smithi* | 92.6 |
| 16 | B4Trans68 | *S. mesaensis* | B | *Sphingomonas paucimobilis* | 99.7 |
| 32 | B4Trans71 | *S. mesaensis* | B | *Sphingomonas* sp*.* | 98.3 |
| 5 | B4Trans72 | *S. mesaensis* | B | Uncultured bacterium clone L4 1Scorpion *Mycoplasma -V. smithi* | 98.8 |
| 33 | 29879_62 | *S. mesaensis* | C | *Bacillus* sp*.* | 95.6 |
| 8 | 29879_63 | *S. mesaensis* | C | *Bacillus niacini* | 95.3 |
| 6 | 29879_64 | *S. mesaensis* | C | *Acidovorax avenae* | 98.0 |
| 24 | 29879_65 | *S. mesaensis* | C | Uncultured *actinobacterium* | 90.9 |
| 3 | 30271_16 | *S. mesaensis* | C | *Escherichia fergusonii* | 99.3 |
| 3 | 30271_17 | *S. mesaensis* | C | *Escherichia fergusonii* | 98.7 |
| 3 | 30271_18 | *S. mesaensis* | C | *Escherichia coli* | 98.3 |
| 19 | 30271_19 | *S. mesaensis* | C | *Leptolyngbya subtilissima* | 98.4 |
| 26 | 30271_20 | *S. mesaensis* | C | *Luteitalea pratensis* | 88.3 |
| 23 | 30271_21 | *S. mesaensis* | C | *Chryseobacterium* sp. | 99.1 |
| 3 | B4Trans35 | *S. mesaensis* | C | *Escherichia coli* | 99.7 |
| 3 | B4Trans67 | *S. mesaensis* | C | *Escherichia fergusonii* | 99.5 |
| 3 | C4Trans21 | *S. mesaensis* | C | *Escherichia coli* | 97.8 |
| 15 | 29879_61 | *S. mesaensis* | D | *Streptomyces kanamyceticus* | 94.3 |

The table shows the bacterial sequences identified from scorpions, according to the top result with a named relative from the BLAST search using the NCBI database. The code indicates the sequencing run (30269) followed by the sample number (10). Some codes for *S. mesaensis* indicate the scorpion (A) and its telson (4) followed by the transformant number (Trans64). The scorpion species and scorpion identifiers are - *Hadrurus arizonensis* (1-7; n = 7) and *Smeringurus mesaensis* (A-D; n = 4). The sequences are ordered by species. “No analysis” indicates sequences that were too short in the parameters defined in mothur for OTU clustering.
